# Increased serum fibroblast growth factor 2 levels, altered peripheral blood inflammatory indicators and their ratios in amyotrophic lateral sclerosis: a clinical exploratory study

**DOI:** 10.3389/fneur.2026.1859903

**Published:** 2026-08-18

**Authors:** Xiaoting Niu, Meijuan Zhang, Huanjie Huang, Bei Shao, Xun Wang, Zhengyi Cai

**Affiliations:** 1 Department of Neurology, The First Affiliated Hospital of Wenzhou Medical University, Wenzhou, Zhejiang, China; 2 Department of Clinical Laboratory, The First Affiliated Hospital of Wenzhou Medical University, Wenzhou, Zhejiang, China

**Keywords:** amyotrophic lateral sclerosis, clinical exploratory study, fibroblast growth factor 2, peripheral blood inflammatory indicators, ratio

## Abstract

**Introduction:**

Amyotrophic lateral sclerosis (ALS) is a progressive neurodegenerative disease characterized by motor neuron degeneration, leading to muscle weakness, atrophy, and ultimately respiratory failure. Previous research has highlighted the roles of neurotrophic factors and inflammatory responses in ALS pathogenesis; however, their interplay remains poorly understood. This exploratory study aims to elucidate the expression characteristics and correlations of fibroblast growth factor 2 (FGF2) and peripheral blood inflammatory indicators (PBIIs) such as derived neutrophil-to-lymphocyte ratio (dNLR) and systemic inflammatory response index (SIRI) in ALS patients.

**Methods:**

This was a prospective case–control study, involving ALS patients meeting Gold Coast diagnostic criteria and age- and sex-matched healthy controls (HCs). Serum FGF2 levels were measured using enzyme-linked immunosorbent assay (ELISA), and PBIIs were assessed through routine blood analysis. Statistical analysis was conducted mainly using intergroup comparison, correlation analysis, and multivariate linear regression.

**Results:**

The results showed significantly elevated levels of FGF2 and PBIIs in ALS patients compared to HCs, with strong correlations between FGF2, PBIIs, clinical staging, and disease progression rates. In multivariate linear regression analysis, PBIIs, especially SIRI, were significantly associated with disease severity and early disease progression. While serum FGF2 and FGF2 to dNLR ratio demonstrated potential as an auxiliary biomarker for later-stage disease.

**Discussion:**

These findings provide new insights into the molecular mechanisms underlying ALS and suggest practical diagnostic and prognostic tools, reinforcing the importance of targeting neurotrophic and inflammatory pathways in ALS management. This study holds significant promise for advancing both clinical practice and future research endeavors in the field.

## Introduction

Amyotrophic lateral sclerosis (ALS) is a devastating neurodegenerative disorder characterized by the progressive degeneration of upper and lower motor neurons, leading to relentless clinical decline and, ultimately, respiratory failure (1). Despite accounting for only a small fraction of neurological diseases, ALS exerts a disproportionate impact due to its rapid progression and high mortality. Patients experience not only profound motor impairment but also secondary complications that severely compromise quality of life. The disease imposes a significant psychological and economic burden on patients, their families, and the wider healthcare system, highlighting the urgent need for improved diagnostic and therapeutic strategies. The majority of cases are sporadic, with only 5–10% attributed to familial or monogenic forms, underscoring the complex interplay between genetic and environmental influences in disease etiology (2). The median survival following diagnosis remains dismally short, typically ranging from 2 to 5 years, further emphasizing the need for advances in early detection and disease monitoring.

Current clinical management of ALS is hampered by significant diagnostic challenges. The absence of disease-specific biomarkers means that diagnosis relies heavily on clinical evaluation and exclusion of mimicking conditions, often necessitating electromyographic and neuroimaging studies (3). This reliance on clinical features contributes to a diagnostic delay of up to 16 months from symptom onset, thereby restricting opportunities for early intervention and inclusion in clinical trials (4). Although several putative biomarkers have been explored—including neurofilament light chain, TAR DNA binding protein 43 (TDP-43), and inflammatory mediators—none have yet demonstrated sufficient sensitivity or specificity to be adopted into routine clinical practice (5). Furthermore, current therapeutic options, such as riluzole and edaravone, provide only modest benefit, highlighting the limitations of existing strategies and the imperative to identify novel molecular targets and reliable biomarkers for ALS (1, 4, 5).

A growing body of literature implicates both neuroinflammatory processes and disruptions of neurotrophic support in the pathogenesis and progression of ALS. Research has identified aberrant activation of peripheral and central immune pathways, resulting in elevated levels of pro-inflammatory cytokines and immune cell activation in patients with ALS (6, 7). Simultaneously, deficits in neurotrophic factors, such as brain-derived neurotrophic factor (BDNF) and fibroblast growth factor 2 (FGF2), have been associated with increased neuronal vulnerability and impaired reparative mechanisms (8). While these two pathological axes—neuroinflammation and neurotrophic dysregulation—are each recognized contributors to motor neuron degeneration, their potential interplay remains insufficiently characterized (9, 10). Recent studies suggest that inflammatory responses may exacerbate neuronal injury, while neurotrophic factors could modulate inflammatory cascades, yet systematic investigation of their interactions in ALS is lacking (11, 12).

In light of these gaps, the present study is designed to systematically evaluate the relationship between FGF2, a key neurotrophic factor, and peripheral blood inflammatory indicators (PBIIs) in ALS. Prior investigations have examined individual roles of growth factors and inflammatory markers, but comprehensive analyses exploring how their dynamic balance might reflect or influence disease stage and progression are still absent. Notably, the ratio of neurotrophic to inflammatory signals in peripheral blood may offer a more nuanced and integrated biomarker than absolute levels alone, yet this hypothesis remains untested in ALS cohorts. By focusing on the FGF2/PBIIs axis and its relevance to clinical staging and disease trajectory, this study addresses a critical knowledge void and has the potential to inform both mechanistic understanding and biomarker discovery.

The methodology employed in this research integrates a prospective, case–control design, including ALS patients diagnosed according to the Gold Coast criteria (13) and age- and sex-matched healthy controls. Serum FGF2 concentrations are quantified using enzyme-linked immunosorbent assay (ELISA), ensuring high sensitivity and reproducibility, while PBIIs are assessed via standardized hematological techniques. Additionally, detailed clinical profiling enables correlation of molecular findings with established functional and staging metrics. The statistical approach leverages multivariate regression analyses to delineate associations between biomarkers and clinical parameters, as well as to interrogate the diagnostic and prognostic performance of the FGF2 to PBIIs ratio. This combination of rigorous clinical phenotyping, robust biomarker measurement, and advanced analytics is designed to maximize the translational value of the findings.

Ultimately, the aims of this clinical exploratory study are threefold: first, to characterize differences in serum FGF2 and PBIIs between ALS patients and healthy individuals; second, to elucidate associations between these biomarkers and key clinical features of ALS, including disease stage, severity, and progression rate; and third, to assess the potential utility of the FGF2 to PBIIs ratio as a candidate biomarker for early diagnosis and disease monitoring. The adoption of minimally invasive, easily repeatable blood-based assays underscores the clinical feasibility and potential for rapid translation into practice. Through these efforts, the study seeks to advance the field toward more precise molecular stratification of ALS and to contribute to the development of improved diagnostic and prognostic tools.

## Methods

### Subjects

This was a prospective study conducted in the First Affiliated Hospital of Wenzhou Medical University, from January to August in 2025. The study was approved by Ethics Committee in Clinical Research of the First Affiliated Hospital of Wenzhou Medical University (Acceptance Number: KY2024-213) and conducted in accordance with all provisions of the Declaration of Helsinki. Written informed consent was obtained from all the participants or their legal guardians.

Patients diagnosed with ALS according to the Gold Coast criteria were enrolled. Patients with severe hepatic and renal failure, autoimmune diseases, active infections, malignancies, and recent use of immunosuppressants or systemic glucocorticoids were excluded. Age- and sex-matched healthy controls (HCs) were recruited from the Medicine and Health Care Center. Age, sex, and body mass index (BMI) were registered for ALS patients and HCs.

For the patients, clinical information was recorded, including site of onset (bulbar or spinal), King stage, age of onset, and disease duration. The disease severity was assessed by the ALS Functional Rating Scale-Revised (ALSFRS-R) score. To evaluate the disease progression rate, the change in ALSFRS-R score (△ALSFRS-R) was calculated as (48 - ALSFRS-R score at enrollment) divided by the duration from symptom onset. Patients were followed up for half a year after enrollment, and ALSFRS-R score were evaluated repeatedly every 3 months if available. ALSFRS-R score^M3-M0^ was calculated as ALSFRS-R score at enrollment - ALSFRS-R score at follow-up of 3 months. ALSFRS-R score^M6-M0^ was calculated as ALSFRS-R score at enrollment - ALSFRS-R score at follow-up of 6 months. ALSFRS-R score^M3-M0^ and ALSFRS-R score^M6-M0^ were applied to evaluated disease progression during follow-up after enrollment.

### Sample collection and laboratory examination

A total of 2 mL serum and 2 mL peripheral blood were collected for analysis. The serum was stored at −80 °C until testing. Serum fibroblast growth factor 2 (FGF2) levels were detected using a commercial ELISA Kit (Cat#DFB50, R&D system). The detection process was strictly performed according to the instructions of the kit. The peripheral blood was sent to the Medical Laboratory Center for routine blood tests after collection, which included determination of blood cell counts such as white blood cells, neutrophils, monocytes, lymphocytes, and platelets. Various peripheral blood inflammatory indicators (PBIIs), including neutrophil-to-lymphocyte ratio (NLR), derived neutrophil-to-lymphocyte ratio (dNLR), monocyte-to-lymphocyte ratio (MLR), neutrophil-to-monocyte-plus-lymphocyte ratio (NMLR), platelet-to-lymphocyte ratio (PLR), systemic immune inflammatory index (SII), systemic inflammatory response index (SIRI), and pan-immune-inflammation value (PIV), were calculated using the following formulas (14):

$$\mathrm{NLR}=\frac{\text{neutrophils}}{\text{lymphocytes}}$$

$$\text{dNLR}=\frac{\text{neutrophils}}{\text{white cell counts}-\text{lymphocytes}}$$

$$\mathrm{MLR}=\frac{\text{monocytes}}{\text{lymphocytes}}$$

$$\text{NMLR}=\frac{\text{monocytes}+\text{neutrophils}}{\text{lymphocytes}}$$

$$\mathrm{PLR}=\frac{\text{platelets}}{\text{lymphocytes}}$$

$$\mathrm{SII}=\frac{\text{platelets}\times \text{neutrophils}}{\text{lymphocytes}}$$

$$\text{SIRI}=\frac{\text{neutrophils}\times \text{monocytes}}{\text{lymphocytes}}$$

$$\mathrm{PIV}=\frac{\text{neutrophils}\times \text{monocytes}\times \text{platelets}}{\text{lymphocytes}}$$

To explore the potential crosstalk between neurotrophic and neuroinflammatory mechanisms, serum FGF2 levels were divided by the above eight indicators (FGF2 to PBIIs ratios).

### Statistical analysis

All statistical analyses were performed using SPSS version 25.0 (IBM Corporation). The distribution of continuous variables was assessed using the Shapiro–Wilk test. Continuous parametric data are presented as mean±standard deviations, while continuous nonparametric data are presented as medians (interquartile range). Based on the distribution assessment, normally distributed variables were compared between two groups using the t-test, while non-normally distributed variables were compared between two groups using the Mann–Whitney U test. Categorical variables were compared between two groups using the Chi-square test. Differences in continuous laboratory biomarkers between the two groups were assessed using the Mann–Whitney U test. To account for multiple comparisons across PBIIs or FGF2 to PBIIs ratios, the False Discovery Rate (FDR) was controlled using the Benjamini-Hochberg procedure. An FDR-adjusted *p* value (q value) < 0.05 was considered statistically significant. The associations between different variables were tested using the Spearman test. Multivariable regression models were established with ALSFRS-R score, ∆ALSFRS-R, ALSFRS-R score^M3-M0^ or ALSFRS-R score^M6-M0^ as dependent variables, while age, site of onset, King stage, disease duration, FGF2, and selected PBIIs were considered as independent variables. To guarantee model reliability, we pre-specified the following criteria for our multivariate linear regression analyses: (1) a minimum of 10 samples per predictor variable to prevent overfitting; (2) for our primary biomarker of interest, a standardized beta coefficient (SBC) (|*β*|) > 0.3 to denote a meaningful effect size; and (3) for model acceptance, both R^2^ and adjusted R^2^ > 0.3 with their difference ≤ 0.1, ensuring substantial explanatory power without overfitting. To further evaluate the risk of overfitting, a post-hoc power analysis was conducted for the regression. Consequently, our final models were parsimonious, including only the core biomarker and the most pertinent clinical confounders. Because of the exploratory nature, nominal *p*-values are reported together with FDR-adjusted q-values; only results with q < 0.05 are considered significant.

Receiver operating characteristic (ROC) curve analysis was performed to evaluate the diagnostic performance of FGF2 and FGF2 to PBIIs ratio. The area under the ROC curve (AUC), sensitivity, and specificity were calculated. The optimal cutoff value was determined by maximizing the Youden index (sensitivity + specificity − 1). The corresponding sensitivity and specificity at the optimal cutoff were reported. A 95% confidence interval (CI) for the AUC was estimated using a nonparametric approach. A *p* value < 0.05 was considered statistically significant.

## Results

### Characteristics of the study population

This study included 30 ALS patients meeting the Gold Coast diagnostic criteria and 30 age- and sex-matched HCs (Table 1). There was no statistical difference in age, sex distribution and BMI between ALS patients and the HCs. Among patients, 13 had bulbar onset, and 17 had spinal onset. Twenty-three patients were at King stages 1 and 2, and 7 patients were at King stages 3 to 5. Age at onset of patients was 63.6 ± 9.6 years; the median disease duration was 15.0 months. The median ALSFRS-R score at enrollment was 41.5, and disease progression rate (△ALSFRS-R) was 0.6. At the 3-month follow-up, 4 patients were lost to follow-up and 2 patients died; at the 6-month follow-up, 2 patients died. At the time of sample collection, five patients had received riluzole monotherapy, while three patients were treated with a combination of riluzole and edaravone. In addition, some patients had chronic conditions such as hypertension and diabetes, for which they had been taking relevant medications on a long-term, regular basis. Based on clinical experience, these medications were unlikely to have a substantial impact on the assay results; therefore, they were not listed.

**Table 1:** Comparison of general information and laboratory results between ALS patients and HC.

Parameters	ALS (*n* = 30)	HC (*n* = 30)	*P* value	*q* value
Age (year)	64.9 ± 9.2	60.9 ± 6.45	0.055^a^	-
Male/Female (n)	16/14	13/17	0.438^b^	-
BMI	22.51 ± 3.37	24.02 ± 2.61	0.056^a^	-
Bulbar/Spinal onset (n)	13/17	-	-	-
King stage 1–2/3–5 (n)	23/7	-	-	-
Age of onset (year)	63.6 ± 9.6	-	-	-
Disease duration (month)	15.0 (6.8–24.0)	-	-	-
ALSFRS-R score	41.5 (33.5–44.3)	-	-	-
△ALSFRS-R^0^	0.6 (0.4–1.0)	-	-	-
Serum FGF2 (pg/ml)	10.64 (7.00–14.15)	3.92 ± 1.26	<0.001^c^	**-**
White cell count (×10^9^/L)	6.22 ± 1.42	5.52 ± 1.29	0.052^a^	-
Neutrophils (×10^9^/L)	3.92 (3.16–5.46)	3.19 ± 0.83	0.002^c^	-
Monocytes (×10^9^/L)	0.37 ± 0.09	0.33 ± 0.12	0.158^a^	-
Lymphocytes (×10^9^/L)	1.52 ± 0.49	1.80 ± 0.48	0.032^a^	-
Platelets (×10^9^/L)	229.83 ± 57.84	212.87 ± 37.27	0.182^a^	-
NLR	2.34 (2.07–3.87)	1.81 (1.49–2.08)	<0.001^c^	<0.001
dNLR	0.90 (0.86–0.92)	0.86 ± 0.04	0.003^c^	0.004
MLR	0.27 (0.19–0.33)	0.19 ± 0.06	0.001^c^	0.001
NMLR	2.53 (2.23–4.14)	1.97 (1.65–2.27)	<0.001^c^	<0.001
PLR	160.21 ± 47.09	126.04 ± 35.98	0.003^c^	0.004
SII	612.35 (460.28–810.62)	394.58 ± 132.56	<0.001^c^	<0.001
SIRI	0.90 (0.69–1.50)	0.63 ± 0.31	<0.001^c^	<0.001
PIV	204.85 (159.27–338.83)	117.43 (61.72–187.86)	<0.001^c^	<0.001
FGF2 to NLR ratio	4.86 (2.29–5.85)	2.15 (1.74–2.84)	<0.001^c^	<0.001
FGF2 to dNLR ratio	12.16 (7.77–16.81)	4.58 ± 1.50	<0.001^c^	<0.001
FGF2 to MLR ratio	41.74 (25.07–59.56)	20.82 (17.57–26.55)	<0.001^c^	<0.001
FGF2 to NMLR ratio	4.40 (2.14–5.20)	1.95 (1.58–2.56)	<0.001^c^	<0.001
FGF2 to PLR ratio	0.07 (0.05–0.10)	0.03 ± 0.01	<0.001^c^	<0.001
FGF2 to SII ratio	0.02 ± 0.01	0.01 ± 0.00	0.002^c^	0.003
FGF2 to SIRI ratio	10.54 (6.20–16.72)	6.55 (4.59–9.99)	0.023^c^	0.025
FGF2 to PIV ratio	0.05 (0.03–0.08)	0.03 (0.02–0.06)	0.062^c^	0.062

ALS, amyotrophic lateral sclerosis; HC, healthy control; BMI, body mass index; ALSFRS-R, ALS Functional Rating Scale-Revised; △ALSFRS-R^0^, (48- ALSFRS-R score) at enrollment/ duration from symptom onset; FGF2, fibroblast growth factor acidic 2; NLR, neutrophil-to-lymphocyte ratio; dNLR, derived neutrophil-to-lymphocyte ratio; MLR, monocyte-to-lymphocyte ratio; NMLR, neutrophil-to-monocyte-plus-lymphocyte ratio; PLR, platelet-to-lymphocyte ratio; SII, systemic immune inflammatory index; SIRI, systemic inflammatory response index; PIV, pan-immune-inflammation value.

a *t*-test.

b Chi-square test.

c Mann-Whitney U test.

### Comparison of serum FGF2 level, PBIIs and FGF2 to PBIIs ratios

The serum FGF2 level in ALS patients was significantly higher than the HCs (Table 1). The comparison results of peripheral blood routine parameters and PBIIs between ALS patients and HCs are also shown in Table 1. Compared to the HCs, ALS patients had significantly increased neutrophil counts and decreased lymphocyte counts. There were no significant differences in white cell count, monocytes, and platelets between the two groups. ALS patients showed significantly higher levels of all the PBIIs compared to the HCs (Table 1). Compared to HCs, ALS patients showed significantly higher all of the FGF2 to PBIIs ratios except for FGF2 to PIV ratio (Table 1).

### Correlation analysis of laboratory indicators and clinical characteristics in ALS patients

**Figure 1:**
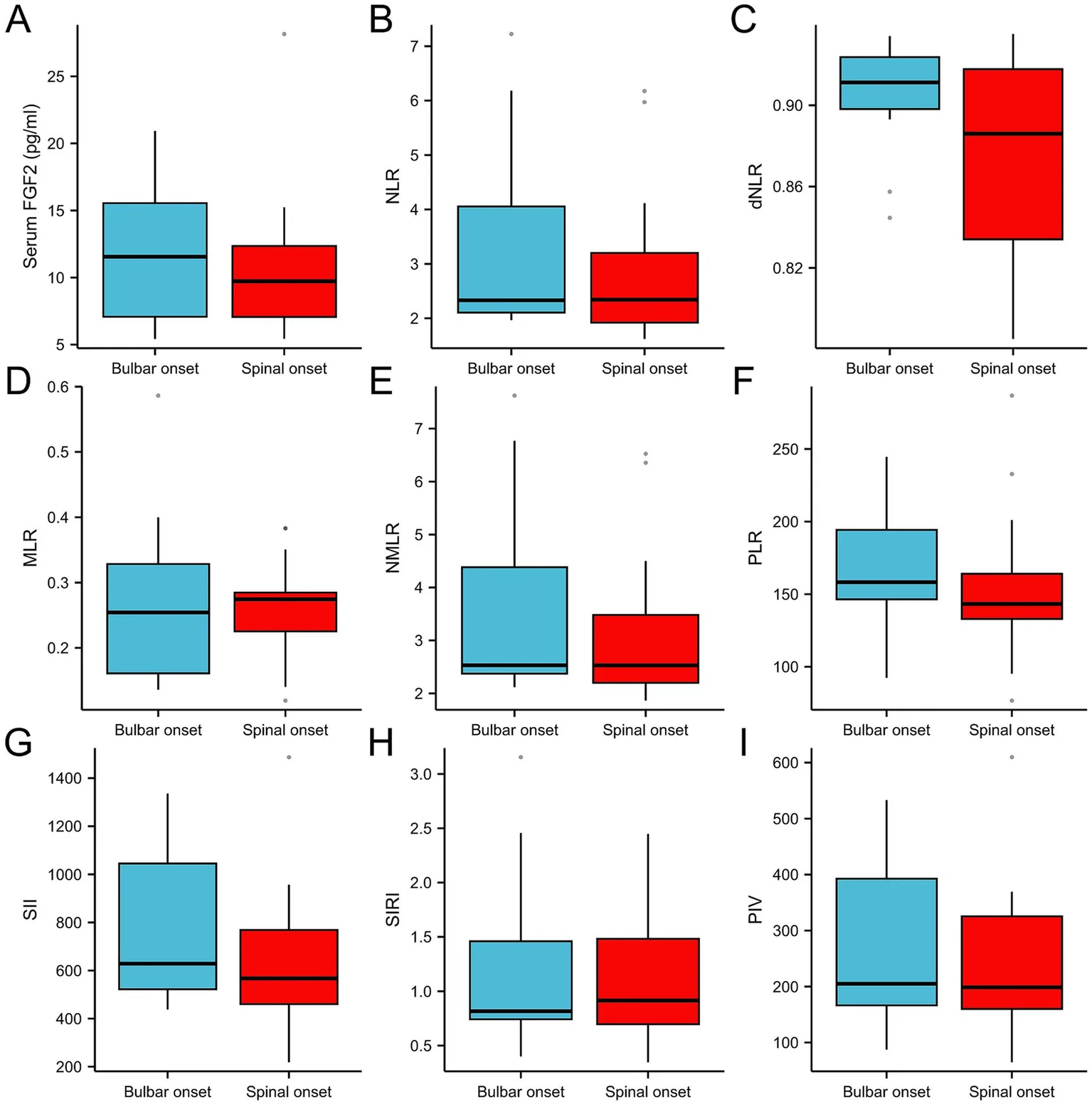
Comparison of serum FGF2 and PBIIs between ALS patients with bulbar onset and spinal onset. **(A)** FGF2, **(B)** NLR, **(C)** dNLR, **(D)** MLR, **(E)** NMLR, **(F)** PLR, **(G)** SII, **(H)** SIRI, **(I)** PIV. FGF2: fibroblast growth factor 2, NLR: neutrophil-to-lymphocyte ratio, dNLR: derived neutrophil-to-lymphocyte ratio, MLR: monocyte-to-lymphocyte ratio, NMLR: neutrophil-to-monocyte-plus-lymphocyte ratio, PLR: platelet-to-lymphocyte ratio, SII: systemic immune inflammatory index, SIRI: systemic inflammatory response index, PIV: pan-immune-inflammation value.

**Figure 2:**
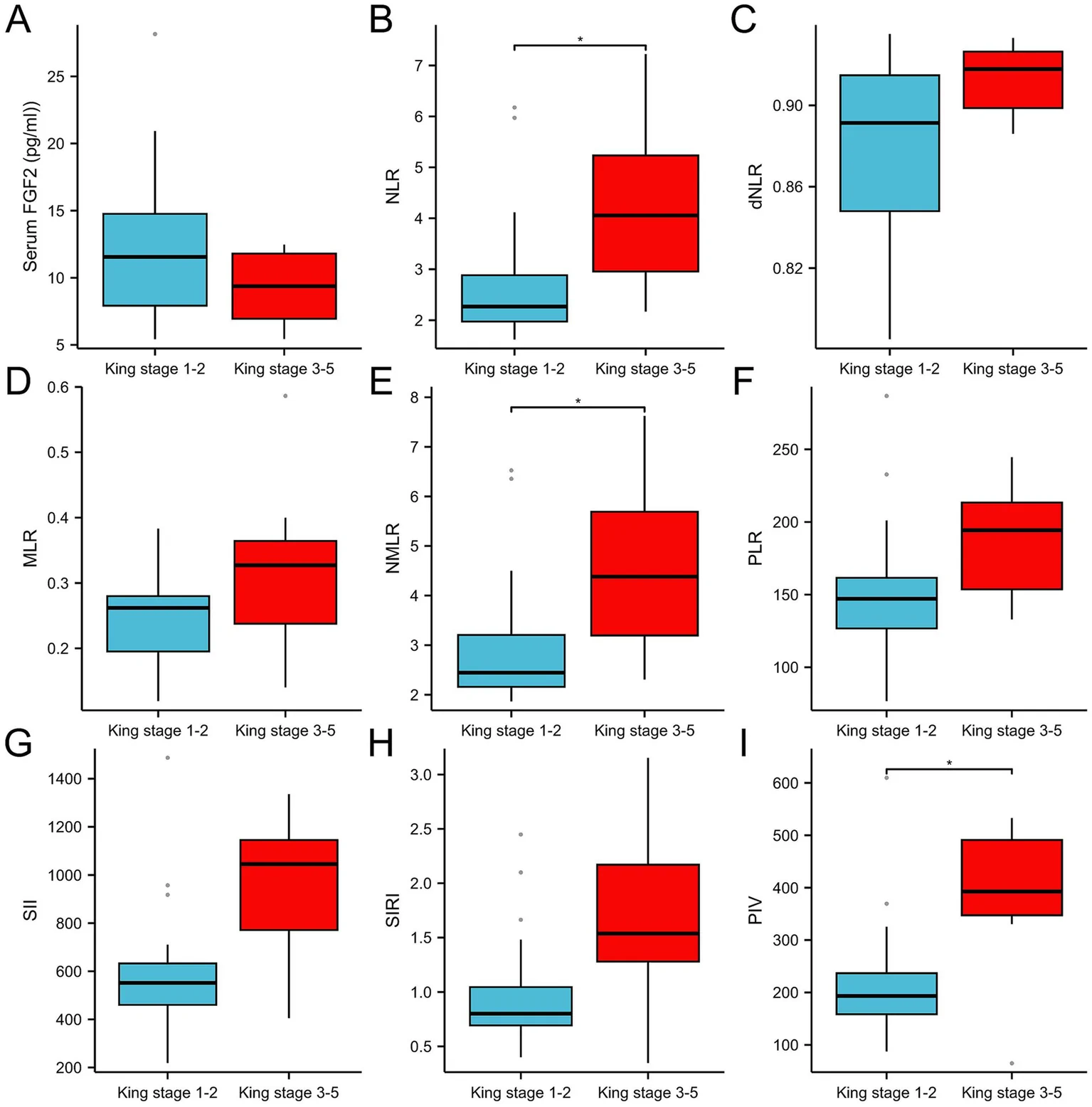
Comparison of serum FGF2 and PBIIs between ALS patients with King stage 1-2 and King stage 3-5. **(A)** FGF2, **(B)** NLR, **(C)** dNLR, **(D)** MLR, **(E)** NMLR, **(F)** PLR, **(G)** SII, **(H)** SIRI, **(I)** PIV. FGF2: fibroblast growth factor 2, NLR: neutrophil-to-lymphocyte ratio, dNLR: derived neutrophil-to-lymphocyte ratio, MLR: monocyte-to-lymphocyte ratio, NMLR: neutrophil-to-monocyte-plus-lymphocyte ratio, PLR: platelet-to-lymphocyte ratio, SII: systemic immune inflammatory index, SIRI: systemic inflammatory response index, PIV: pan-immune-inflammation value.

**Figure 3:**
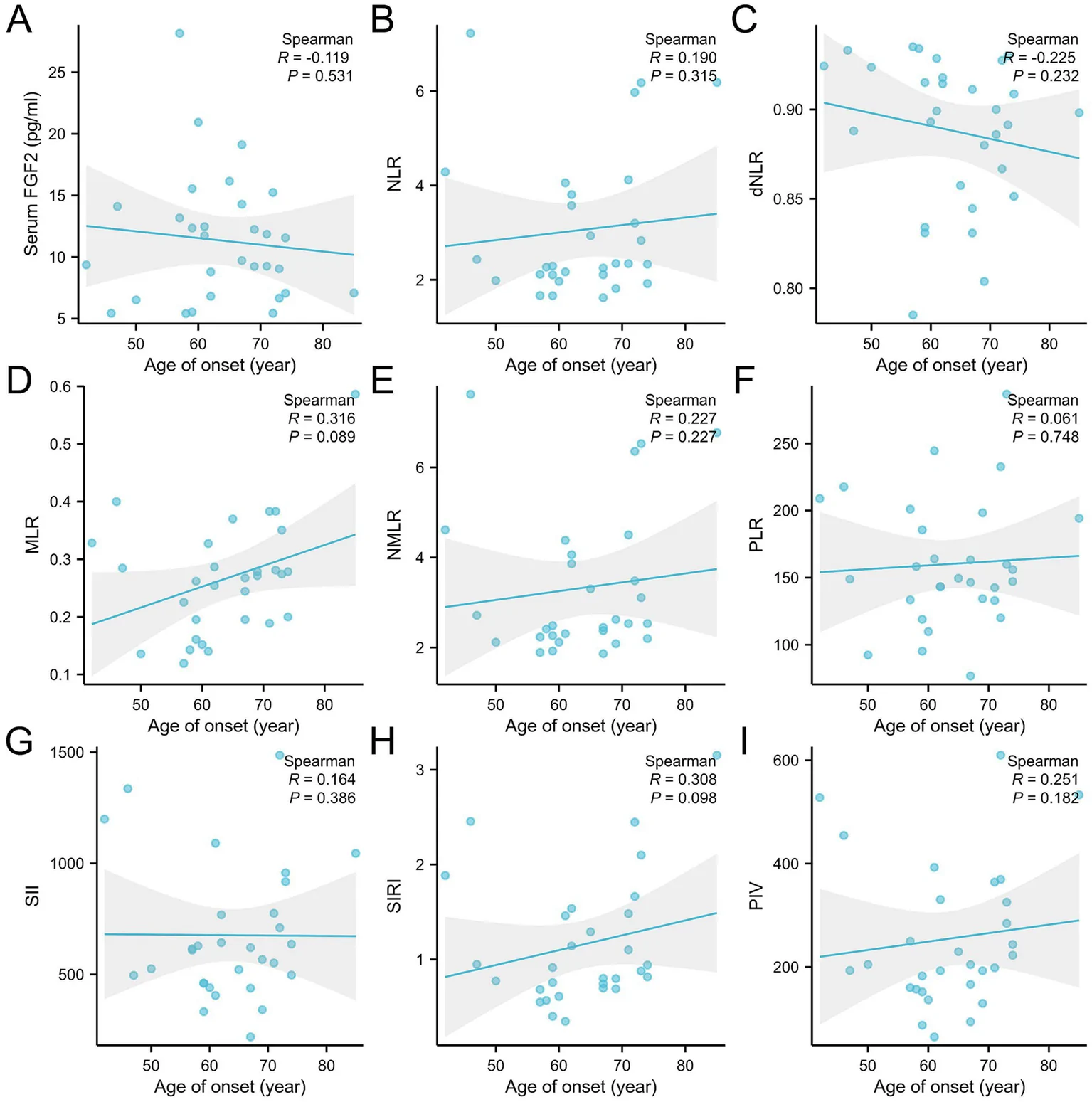
Correlation analysis of laboratory indicators and age of onset in ALS patients. **(A)** FGF2, **(B)** NLR, **(C)** dNLR, **(D)** MLR, **(E)** NMLR, **(F)** PLR, **(G)** SII, **(H)** SIRI, **(I)** PIV. FGF2: fibroblast growth factor 2, NLR: neutrophil-to-lymphocyte ratio, dNLR: derived neutrophil-to-lymphocyte ratio, MLR: monocyte-to-lymphocyte ratio, NMLR: neutrophil-to-monocyte-plus-lymphocyte ratio, PLR: platelet-to-lymphocyte ratio, SII: systemic immune inflammatory index, SIRI: systemic inflammatory response index, PIV: pan-immune-inflammation value.

**Figure 4:**
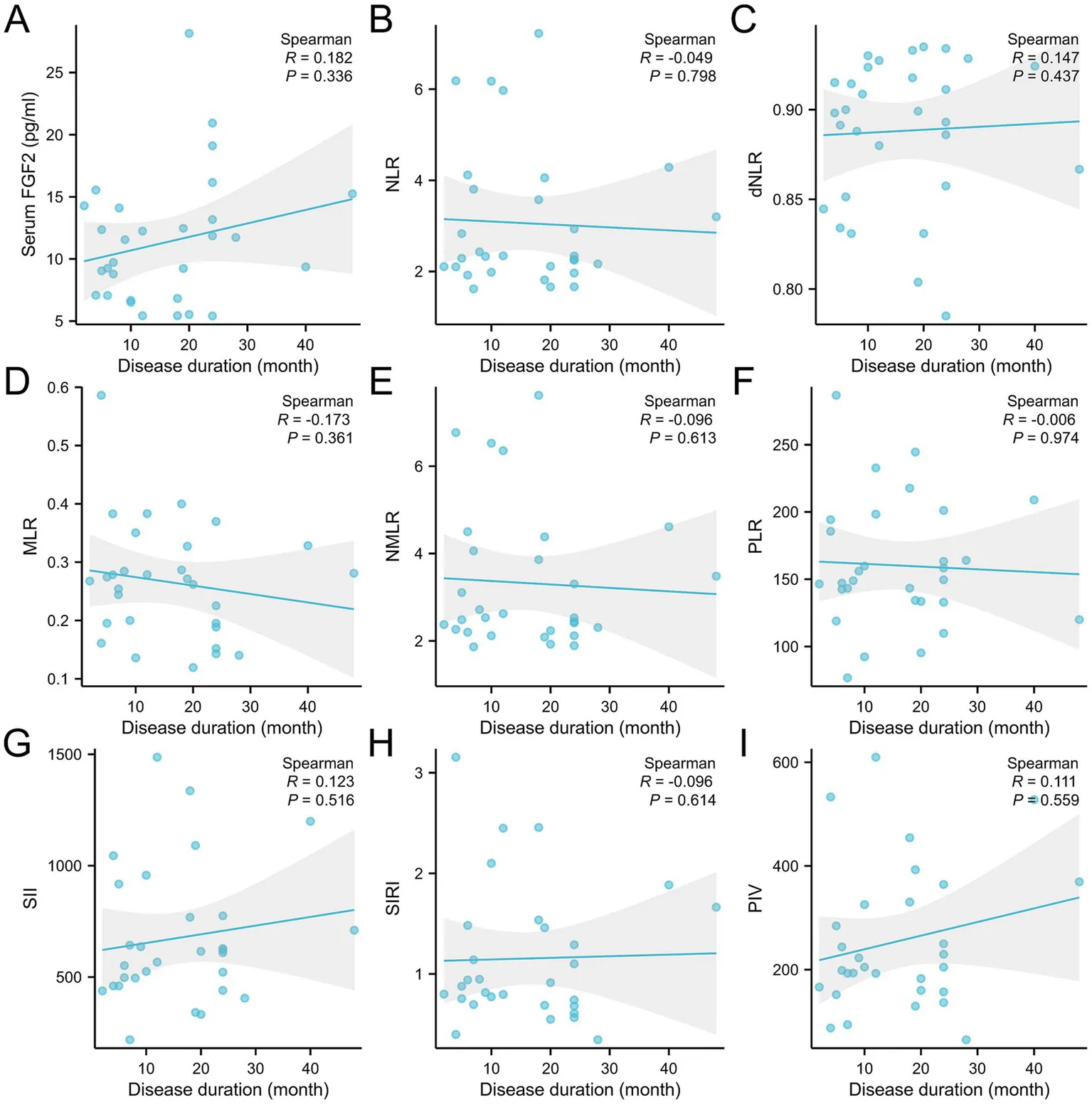
Correlation analysis of laboratory indicators and disease duration in ALS patients. **(A)** FGF2, **(B)** NLR, **(C)** dNLR, **(D)** MLR, **(E)** NMLR, **(F)** PLR, **(G)** SII, **(H)** SIRI, **(I)** PIV. FGF2: fibroblast growth factor 2, NLR: neutrophil-to-lymphocyte ratio, dNLR: derived neutrophil-to-lymphocyte ratio, MLR: monocyte-to-lymphocyte ratio, NMLR: neutrophil-to-monocyte-plus-lymphocyte ratio, PLR: platelet-to-lymphocyte ratio, SII: systemic immune inflammatory index, SIRI: systemic inflammatory response index, PIV: pan-immune-inflammation value.

**Figure 5:**
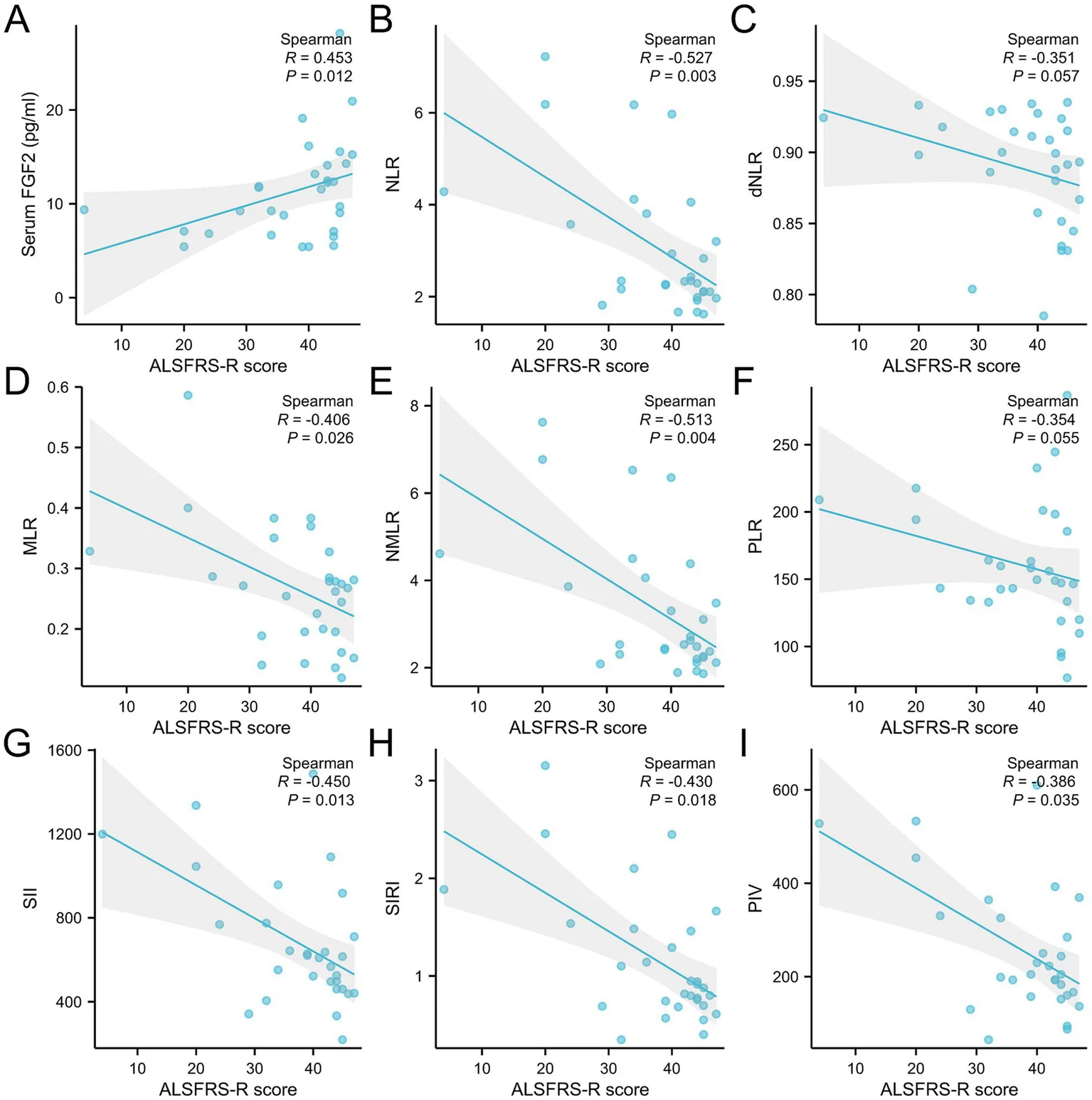
Correlation analysis of laboratory indicators and ALSFRS-R score in ALS patients. **(A)** FGF2, **(B)** NLR, **(C)** dNLR, **(D)** MLR, **(E)** NMLR, **(F)** PLR, **(G)** SII, **(H)** SIRI, **(I)** PIV. FGF2: fibroblast growth factor 2, NLR: neutrophil-to-lymphocyte ratio, dNLR: derived neutrophil-to-lymphocyte ratio, MLR: monocyte-to-lymphocyte ratio, NMLR: neutrophil-to-monocyte-plus-lymphocyte ratio, PLR: platelet-to-lymphocyte ratio, SII: systemic immune inflammatory index, SIRI: systemic inflammatory response index, PIV: pan-immune-inflammation value.

**Figure 6:**
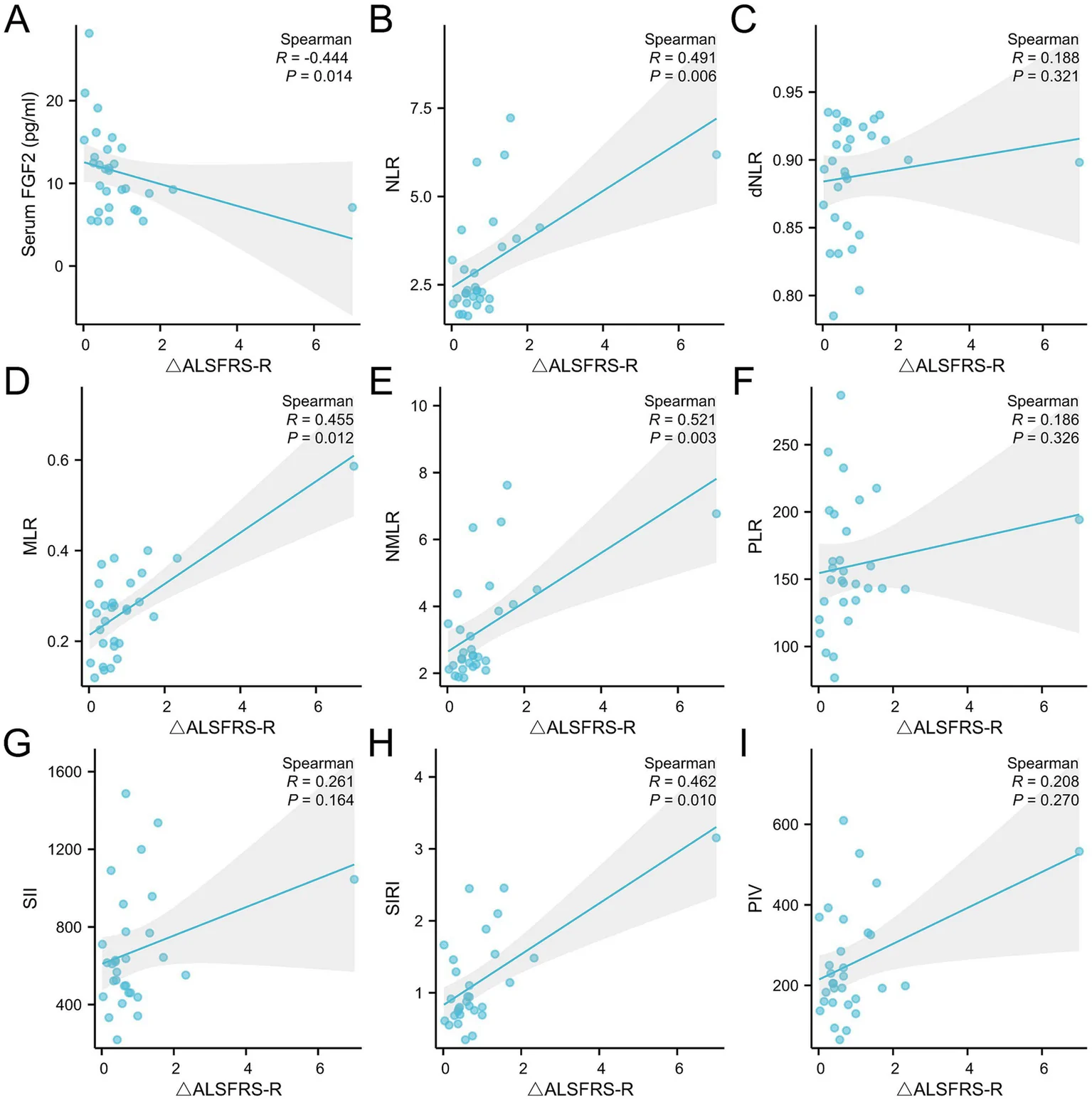
Correlation analysis of laboratory indicators and △ ALSFRS-R in ALS patients. **(A)** FGF2, **(B)** NLR, **(C)** dNLR, **(D)** MLR, **(E)** NMLR, **(F)** PLR, **(G)** SII, **(H)** SIRI, **(I)** PIV. FGF2: fibroblast growth factor 2, NLR: neutrophil-to-lymphocyte ratio, dNLR: derived neutrophil-to-lymphocyte ratio, MLR: monocyte-to-lymphocyte ratio, NMLR: neutrophil-to-monocyte-plus-lymphocyte ratio, PLR: platelet-to-lymphocyte ratio, SII: systemic immune inflammatory index, SIRI: systemic inflammatory response index, PIV: pan-immune-inflammation value.

There were no statistically significant differences in serum FGF2 levels or PBIIs between ALS patients with two onset forms (Figure 1). NLR (Figure 2B), NMLR (Figure 2E), and PIV (Figure 2I) were significantly higher in patients with King stages 3 to 5 (q < 0.05). There were no significant differences in serum FGF2 (Figure 2A) and other indicators such as dNLR (Figure 2C), MLR (Figure 2D), PLR (Figure 2F), SII (Figure 2G), SIRI (Figure 2H) between different King stages, although serum FGF2 levels tended to decrease and indicators increased with advancing stage. Correlation analysis found no significant associations between any parameters and age at onset (Figure 3) or disease duration (Figure 4). Serum FGF2 levels were significantly positively correlated with ALSFRS-R scores (Figure 5A), and significantly negatively correlated with ∆ALSFRS-R (Figure 6A). All the PBIIs except for PLR were significantly negatively correlated with ALSFRS-R scores (Figures 5B–I), while only NLR (Figure 6B), MLR (Figure 6D), NMLR (Figure 6E), and SIRI (Figure 6H) were significantly positively correlated with ∆ALSFRS-R.

### Correlation between different laboratory indicators

In ALS patients, serum FGF2 levels were significantly positively correlated with the SIRI (Supplementary Figure 1G). In HCs, there was no significant correlation between serum FGF2 levels and any PBIIs (Supplementary Figure 2).

### The analysis of serum FGF2 to PBIIs ratios

There were no statistically significant differences in ratios between ALS patients with different onset forms (Supplementary Figure 3) and King stage (Supplementary Figure 4). Correlation analysis found no significant associations between any ratios and age at onset (Supplementary Figure 5) or disease duration (Supplementary Figure 6). All of the ratios were positively correlated with ALSFRS-R scores (Supplementary Figure 7), while significantly negatively correlated with ∆ALSFRS-R,except for FGF2 to PIV ratio (Supplementary Figure 8).

### Multivariate linear regression model for predictors of disease severity and progression

Based on the previously established model screening rules, a total of 25 meaningful multiple linear models were screened out (Table 2). Overall, the King stage is an indicator closely related to the ALSFRS-R score. The SIRI showed statistical significance in various models with the ALSFRS-R score and ∆ALSFRS-R as dependent variables, suggesting that this indicator is related to disease severity and early disease progression. FGF2 and the FGF2 to dNLR ratio showed statistical significance in various models with the ALSFRS-R score^M3-M0^ and ALSFRS-R score^M6-M0^ as dependent variables, indicating that these two indicators may be associated with later-stage disease progression.

**Table 2:** Multivariate linear regression model for predictors of ALSFRS-R score, ∆ALSFRS-R, ALSFRS-R score^M3-M0^ and ALSFRS-R score^M6-M0^.

Dependent variable	Model parameters				Independent variable 1					Independent variable 2					Independent variable 3					
	R^2^	Adjust R^2^	*P* value	PV	Name	SBC	Lower CI	Upper CI	*P* value	Name	SBC	Lower CI	Upper CI	*P* value	Name		SBC	Lower CI	Upper CI	*P* value
ALSFRS-R score	0.629	0.586	<0.001	1.000	King stage	−0.511	−18.450	−5.153	0.001	Age of onset	0.202	−0.064	0.483	0.127	SIRI		−0.388	−9.951	−1.405	0.011
	0.599	0.552	<0.001	1.000	King stage	−0.589	−20.048	−7.143	<0.001	Age of onset	0.145	−0.123	0.425	0.268	FGF2 to NLR ratio		0.302	0.079	1.976	0.035
	0.599	0.553	<0.001	1.000	King stage	−0.594	−20.117	−7.298	<0.001	Age of onset	0.146	−0.122	0.426	0.264	FGF2 to NMLR ratio		0.301	0.088	2.123	0.034
	0.603	0.557	<0.001	1.000	King stage	−0.538	−19.342	−5.503	0.001	Disease duration	−0.105	−0.336	0.147	0.427	SIRI		−0.329	−9.015	−0.601	0.027
	0.597	0.551	<0.001	1.000	King stage	−0.565	−19.821	−6.292	0.001	Disease duration	−0.147	−0.383	0.117	0.284	FGF2 to NLR ratio		0.311	0.092	2.019	0.033
	0.598	0.551	<0.001	1.000	King stage	−0.570	−19.893	−6.456	<0.001	Disease duration	−0.148	−0.384	0.117	0.283	FGF2 to NMLR ratio		0.309	0.101	2.168	0.033
	0.465	0.403	0.001	0.986	Age of onset	0.283	−0.034	0.623	0.077	Disease duration	−0.170	−0.433	0.126	0.268	SIRI		−0.634	−13.730	−4.840	<0.001
	0.385	0.314	0.005	0.936	Age of onset	0.185	−0.152	0.536	0.262	Disease duration	−0.288	−0.560	0.039	0.085	FGF2 to NLR ratio		0.555	0.796	2.977	0.001
	0.378	0.306	0.006	0.929	Age of onset	0.187	−0.152	0.541	0.260	Disease duration	−0.291	−0.565	0.038	0.085	FGF2 to NMLR ratio		0.549	0.831	3.200	0.002
∆ALSFRS-R	0.525	0.471	<0.001	0.997	King stage	0.200	−0.370	1.542	0.219	Age of onset	0.309	0.002	0.080	0.042	SIRI		0.499	0.315	1.543	0.005
	0.487	0.428	0.001	0.992	King stage	0.321	−0.060	1.947	0.064	Disease duration	−0.410	−0.083	−0.012	0.011	NLR		0.401	0.060	0.600	0.018
	0.604	0.558	<0.001	1.000	King stage	0.248	−0.151	1.607	0.101	Disease duration	−0.423	−0.079	−0.018	0.003	SIRI		0.556	0.501	1.569	<0.001
	0.481	0.421	0.001	0.991	Age of onset	0.271	−0.004	0.076	0.078	Disease duration	−0.238	−0.062	0.007	0.118	NLR		0.524	0.191	0.671	0.001
	0.492	0.434	<0.001	0.993	Age of onset	0.262	−0.005	0.074	0.085	Disease duration	−0.236	−0.062	0.007	0.117	NMLR		0.536	0.193	0.647	0.001
	0.583	0.535	<0.001	1.000	Age of onset	0.165	−0.015	0.059	0.235	Disease duration	−0.308	−0.067	−0.004	0.028	SIRI		0.630	0.673	1.671	<0.001
	0.402	0.333	0.003	0.951	Age of onset	0.236	−0.012	0.075	0.154	Disease duration	−0.367	−0.081	−0.004	0.033	PIV		0.457	0.001	0.007	0.008
ALSFRS-R score^M3-M0^	0.412	0.324	0.012	0.959	King stage	−0.180	−4.806	1.698	0.331	Age of onset	0.243	−0.040	0.192	0.188	FGF2		−0.558	−0.562	−0.113	0.005
	0.439	0.355	0.008	0.976	King stage	−0.202	−4.942	1.456	0.269	Age of onset	0.242	−0.038	0.189	0.180	FGF2 to dNLR ratio		−0.587	−0.515	−0.120	0.003
	0.411	0.322	0.013	0.959	King stage	−0.219	−5.177	1.399	0.245	Disease duration	−0.172	−0.160	0.061	0.360	FGF2 to dNLR ratio		−0.573	−0.520	−0.101	0.006
	0.404	0.315	0.014	0.953	Age of onset	0.237	−0.046	0.194	0.211	Disease duration	−0.158	−0.159	0.067	0.410	FGF2		−0.482	−0.516	−0.067	0.014
	0.421	0.334	0.011	0.965	Age of onset	0.244	−0.042	0.194	0.192	Disease duration	−0.148	−0.154	0.069	0.434	FGF2 to dNLR ratio		−0.501	−0.470	−0.073	0.010
ALSFRS-R score^M6-Mo^	0.446	0.353	0.012	0.979	King stage	−0.321	−6.804	0.646	0.100	Age of onset	0.148	−0.082	0.189	0.421	FGF2		−0.621	−0.681	−0.162	0.003
	0.451	0.359	0.011	0.981	King stage	−0.341	−7.013	0.466	0.082	age of onset	0.154	−0.079	0.190	0.399	FGF2 to dNLR ratio		−0.629	−0.619	−0.150	0.003
	0.447	0.355	0.012	0.980	King stage	−0.318	−6.781	0.688	0.104	Disease duration	−0.156	−0.177	0.075	0.408	FGF2		−0.596	−0.672	−0.137	0.005
	0.448	0.356	0.012	0.980	King stage	−0.339	−7.028	0.518	0.087	Disease duration	−0.148	−0.175	0.078	0.433	FGF2 to dNLR ratio		−0.604	−0.612	−0.126	0.005

ALSFRS-R, ALS Functional Rating Scale-Revised; PV, post-hoc power analysis value; ∆ALSFRS-R, 48 - ALSFRS-R score at enrollment divided by the duration from symptom onset; ALSFRS-R score^M3-M0^, ALSFRS-R score at enrollment - ALSFRS-R score at follow-up of 3 months; ALSFRS-R score^M6-M0^, ALSFRS-R score at enrollment - ALSFRS-R score at follow-up of 6 months; SBC, standardized beta coefficient; CI, confidence interval; SIRI, systemic inflammatory response index; FGF2, fibroblast growth factor acidic 2; NLR, neutrophil-to-lymphocyte ratio; NMLR, neutrophil-to-monocyte-plus-lymphocyte ratio; dNLR, derived neutrophil-to-lymphocyte ratio.

Benjamini-Hochberg false discovery rate (FDR) correction to the p values across the 25 model showed all FDR-adjusted P value (q-value) were <0.05.

### ROC curve analysis for the diagnostic performance of FGF2 and FGF2 to PBIIs ratio

The ROC analysis for FGF2 yielded an AUC of 0.986 (95% CI: 0.965–1.000, (*p* < 0.001)), with a sensitivity of 96.7% and specificity of 90.0% at the optimal cutoff of 5.43 pg./mL. Among FGF2 to PBIIs ratio, the FGF2 to dNLR ratio showed the best performance, with an AUC of 0.964 (95% CI: 0.926–1.000, (p < 0.001)), sensitivity of 80.0%, and specificity of 100.0% at a cutoff of 7.32. Detailed results are presented in Table 3 and Supplementary Figure 9.

**Table 3:** Diagnostic performance of FGF2 and FGF2 to PBIIs ratio in ALS patients.

Parameters	Cutoff value	Sensitivity (%)	Specificity (%)	Youden index	AUC (95%CI)	*P* value
FGF2	5.43	96.7	90.0	0.870	0.986 (0.965–1.000)	<0.001
FGF2 to NLR ratio	3.16	66.7	93.3	0.600	0.780 (0.654–0.906)	<0.001
FGF2 to dNLR ratio	7.32	80.0	100.0	0.800	0.964 (0.926–1.000)	<0.001
FGF2 to MLR	32.22	70.0	86.7	0.567	0.814 (0.702–0.927)	<0.001
FGF2 to NMLR	2.82	70.0	93.3	0.633	0.784 (0.659–0.910)	<0.001
FGF2 to PLR	0.04	80.0	80.0	0.600	0.876 (0.788–0.963)	<0.001
FGF2 to SII ratio	0.01	60.0	86.7	0.467	0.738 (0.607–0.869)	0.002
FGF2 to SIRI ratio	7.35	73.3	63.3	0.367	0.671 (0.531–0.811)	0.023
FGF2 to PIV ratio	0.03	76.7	46.7	0.233	0.640 (0.500–0.780)	0.062

FGF2, fibroblast growth factor 2; ALS, amyotrophic lateral sclerosis; AUC, area under the curves; CI, confidence interval; NLR, neutrophil-to-lymphocyte ratio; dNLR, derived neutrophil-to-lymphocyte ratio; MLR, monocyte-to-lymphocyte ratio; NMLR, neutrophil-to-monocyte-plus-lymphocyte ratio; PLR, platelet-to-lymphocyte ratio; SII, systemic immune inflammatory index; SIRI, systemic inflammatory response index; PIV, pan-immune-inflammation value.

## Discussion

The present study systematically investigates the alterations in serum FGF2 and PBIIs in individuals with ALS, elucidating their potential interrelationship and clinical relevance. By quantitatively assessing serum FGF2 levels alongside multiple inflammatory parameters, this research delineates their association with disease staging and progression rates. The analysis reveals significant elevations in both FGF2 and inflammatory markers compared to HCs, suggesting a coordinated dysregulation of neurotrophic support and systemic inflammation in ALS pathophysiology. Notably, the derived ratio of FGF2 to PBIIs demonstrates promising correlations with clinical severity indices, intimating its utility as an adjunct biomarker for monitoring disease evolution. Multivariate analysis showed serum FGF2, SIRI, and FGF2 to dNLR ratio may be potential biomarker in monitoring disease severity and progression. ROC curve analysis demonstrated the excellent diagnostic value of FGF2 and the FGF2 to dNLR ratio in ALS patients. These findings provide a compelling framework for understanding the molecular crosstalk underlying ALS and open avenues for biomarker-driven clinical assessment.

A salient finding of this study is the marked elevation of serum FGF2 in ALS patients relative to controls, a result that aligns with several lines of evidence implicating FGF2 as a context-dependent modulator of neurodegeneration and tissue remodeling (15–17). Mechanistically, FGF2 belongs to a family of growth factors involved in neuroprotection, synaptic plasticity, and angiogenesis, but also participates in maladaptive responses such as gliosis and aberrant neuroinflammation under pathological conditions (18, 19). Previous studies have reported both increased and decreased FGF2 levels in distinct disease states, with elevated FGF2 correlating with adverse outcomes in ALS (15, 16). In the neurodegenerative sphere, high plasma FGF2 has been associated with mild cognitive impairment and Alzheimer’s disease, where it may reflect ongoing neuroinflammatory or reparative processes (20). Notably, methodological differences—such as the use of serum versus plasma, assay sensitivity, and patient selection—have contributed to discrepancies in reported FGF2 baseline values and their clinical correlates (21). This study’s demonstration of elevated FGF2 in ALS, particularly when matched against well-characterized controls, supports the hypothesis that FGF2 upregulation may represent a compensatory yet ultimately insufficient response to progressive neuronal injury. This observation also raises the possibility of leveraging FGF2 as a minimally invasive biomarker for ALS diagnosis and disease monitoring, contingent on further standardization of assay techniques and longitudinal validation across diverse patient populations.

The pronounced increase in PBIIs, including NLR, dNLR, MLR, and related composite indices, in ALS patients provides mechanistic insight into the interplay between systemic inflammation and neurodegeneration. Current evidence demonstrates that chronic, low-grade peripheral inflammation is a hallmark of ALS and that specific leukocyte subpopulations are differentially regulated as the disease progresses (22–24). The observed neutrophilia and lymphopenia are consistent with a shift toward innate immune activation and impaired adaptive immunity, a pattern that has been reported in multi-center studies of ALS and is thought to reflect both central and peripheral immune dysregulation (25). Interestingly, the lack of significant differences in total leukocyte count or platelet numbers suggests that the discriminatory value of these indices lies not in absolute cell counts but in the balanced and relative proportions of immune cell subsets. Mechanistically, elevated NLR and related ratios have been proposed to reflect the mobilization of neutrophils in response to tissue damage and the exhaustion or redistribution of lymphocytes, linking peripheral immune status to CNS microglial activation and blood–brain barrier integrity (26). The consistency of these findings with reports from other neuroinflammatory and neurodegenerative conditions further underscores their potential utility as disease activity markers, while the nuances of their regulation in ALS may provide clues to the unique immunopathology of motor neuron degeneration.

The differential expression of FGF2 and PBIIs across King clinical stages, and their robust correlations with ALSFRS-R scores and progression rates, point to a dynamic interplay between neurotrophic signaling and immune-inflammatory processes in modulating disease trajectory. Prior studies in heart failure and neuropsychiatric disorders have established that circulating FGF2 levels are tightly linked to both disease severity and key prognostic parameters, such as ventricular function or symptom burden (27, 28). In ALS, the positive association of FGF2 with functional scores and its inverse relationship with disease progression rate suggest a neuroprotective or compensatory role, while the escalation of PBIIs with advancing clinical stage mirrors the intensification of neuroinflammatory activity documented in histopathological analyses and biomarker studies.

It is currently believed that the pathogenesis of ALS is associated with the abnormal intracellular accumulation of pathological proteins encoded by certain genes, such as SOD1 protein, or TDP-43 protein, have been shown to translocate through extracellular vesicles pathway and subsequently accumulate intracellularly, thus increasing the risk of ALS (3). Aberrant hexanucleotide repeat expansion (HRE) within the *C9orf72* gene results in the generation of five distinct dipeptide repeat proteins (DPRs). Among the DPRs, poly-PR accumulates in the nucleus and exhibits particularly strong toxicity to motor neurons (29). Moreover, p53 pathway activation has been observed with ALS-linked genes, such as mutant *TDP-43* and mutant *SOD1* with DPRs from mutant *C9orf72* strongly associated with p53 activation (29). FGF2 could exhibited neuroprotective effects not only against poly-PR but also poly-GR toxicity (29). Also, FGF2 may contribute to a proinflammatory microenvironment, where neuroprotective autoimmunity prevails (30). From this perspective, it is hypothesized that the increase in FGF2 levels may represent a compensatory response to inflammation and motor neuron injury, intended to suppress the further formation and accumulation of pathological proteins via the activation of certain signaling pathways. Indeed, longitudinal assessment of FGF2 concentrations at different stages of disease progression would provide stronger validation for this hypothesis.

Furthermore, a significant correlation between FGF2 and SIRI was observed in patients with ALS, but not in HCs. Apart from SIRI, FGF2 did not correlate with other PBIIs in HCs. SIRI, which reflects systemic inflammatory response, is derived through a mathematical formula. This finding suggests that in ALS patients, FGF2 may be systemically elevated via interactions with inflammatory mediators or blood cells such as neutrophils, monocytes, or lymphocytes. In HCs, systemic inflammation remains at a very low level, which may account for the absence of such correlations. These results further indicate that the expression of FGF2 remains relatively stable under physiological conditions, and the elevation of FGF2 in ALS likely represents a compensatory response to inflammation.

Notably, while the majority of PBIIs correlate negatively with function and positively with progression, the differential strength and directionality of these associations across specific indices reflect the heterogeneity of immune responses in ALS and the potential influence of comorbidities, medications, or genetic background. This intricate pattern underscores the necessity of integrating multi-marker panels and longitudinal sampling in future prognostic modeling, as single time-point measurements may fail to capture the dynamic evolution of the neuro-immune axis in ALS. Collectively, these findings highlight the interdependence of neurotrophic and inflammatory pathways in ALS progression and suggest that composite biomarker approaches may enhance clinical staging and risk stratification.

The application of multivariate regression and correlation analyses in this study enables a nuanced dissection of the multifactorial determinants of ALS severity and progression. While univariate analyses offer insight into individual biomarker-disease associations, the inclusion of age of onset, clinical staging, disease duration, FGF2, and multiple PBIIs in a multivariate framework allows for the quantification of independent and interactive effects, reducing confounding and enhancing mechanistic inference. Previous studies in diverse clinical contexts, including oncology and metabolic disease, have highlighted the superiority of multivariate statistical models for risk prediction and biomarker evaluation, particularly in the presence of complex inter-variable relationships and potential collinearity. In the context of ALS, this analytical strategy enables the identification of biomarker combinations that most robustly predict functional decline, while also revealing the limitations imposed by small sample sizes and potential overfitting. The findings further reinforce the necessity of multi-center, adequately powered studies to validate and refine multivariate prediction models and to explore the potential for integrating clinical, neuroimaging, and molecular data streams. In summary, the use of advanced statistical modeling elucidates the joint contributions of neurotrophic, inflammatory, and clinical variables to ALS progression, providing a methodological template for future biomarker-driven research in neurodegenerative disease.

Our study identified the FGF2 to PBII ratio as a novel composite biomarker significantly associated with clinical severity in ALS. The strength of this ratio lies in its ability to concurrently reflect two opposing facets of ALS pathophysiology: endogenous neuroprotective-repair processes (represented by FGF2) and systemic inflammatory burden (represented by PBII). The independent inverse association between a lower FGF2/PBII ratio and worse ALSFRS-R score, as demonstrated in our multivariate model, suggests that the functional decline in ALS may be driven not merely by the magnitude of injury, but more critically by a failure of the repair systems to adequately counterbalance ongoing inflammatory damage. Mechanistically, this imbalance could stem from and further exacerbate a vicious cycle involving activated glial cells, increased neuronal vulnerability, and potential dysfunction of the blood-spinal cord barrier. Future studies validating this ratio in larger cohorts and exploring its cellular sources are warranted. Importantly, the FGF2 to PBII ratio, especially FGF2 to dNLR ratio presents a tangible therapeutic target; strategies aimed at concurrently enhancing FGF2 signaling and mitigating PBII-related inflammation may hold promise for restoring this critical equilibrium in ALS.

Certain negative findings warrant further consideration. For instance, the FGF2 to PIV ratio did not significantly differ between ALS patients and HCs. Given that PIV incorporates an additional platelet count component compared with SIRI, it is plausible that platelets do not play a decisive role in the inflammatory response or FGF2 secretion. Alternatively, the lack of statistical significance cannot be excluded due to the limited sample size.

This study is subject to several notable limitations. First, this small single-center exploratory study restricts the statistical power and may increase the risk of type II error, potentially limiting the robustness of the observed associations, especially in subgroup analyses such as onset site and disease stage. There may also be a risk of false negative findings for small effect sizes, and that all multivariate results should be considered exploratory, requiring validation in larger cohort studies. Second, the reliance on single time-point measurements of serum FGF2 and PBIIs preclude the assessment of longitudinal changes and causality, which is critical for elucidating dynamic neuroimmune interactions and for validating the prognostic utility of these biomarkers in ALS progression. Since our findings are correlational, longitudinal studies or experimental models are required to disentangle mechanisms. Third, PBIIs are derived from blood routine tests without confirmation by specific inflammatory cytokines (e.g., interleukins and other inflammatory cytokines). Added detection of key cytokines or more neurotrophic factors such as BDNF and glial cell line-derived neurotrophic factor (GDNF) may help comprehensively corroborate neurotrophic and systemic inflammation status in ALS. Fourth, the 3 to 6 months data are only descriptive of short-term trends, not suitable for long-term prognostic inference. Future studies with longer follow-up and better retention strategies are needed. Fifth, since ALS patients may have been receiving different pharmacological treatments at the time of blood sample collection, the assay results could be influenced accordingly; therefore, caution is warranted when interpreting the findings of the present study. Sixth, given the potential effects of age and sex on immune markers, future studies with larger sample sizes should incorporate demographic variables (including age and sex), clinical characteristics, and hematological parameters into the model to draw more comprehensive conclusions.

Despite these limitations, our findings highlight a significant elevation of serum FGF2 and multiple PBIIs in ALS patients, with their ratios demonstrating strong correlations with disease severity and progression. The novel approach of integrating neurotrophic and inflammatory indices provides valuable insight into the interplay between neuroinflammation and neuroprotection in ALS. Future research should focus on larger, longitudinal cohorts to validate these biomarkers’ prognostic value and clarify their mechanistic roles, ultimately informing the development of targeted interventions and individualized disease monitoring strategies.

## Data Availability

The original contributions presented in the study are included in the article/supplementary material, further inquiries can be directed to the corresponding author.

## Supplementary material

SUPPLEMENTARY FIGURE 1 Correlation analysis of serum FGF2 and PBIIs in ALS patients. NLR: neutrophil-to-lymphocyte ratio, dNLR: derived neutrophil-to-lymphocyte ratio, MLR: monocyte-to-lymphocyte ratio, NMLR: neutrophil-to-monocyte-plus-lymphocyte ratio, PLR: platelet-to-lymphocyte ratio, SII: systemic immune inflammatory index, SIRI: systemic inflammatory response index, PIV: pan-immune-inflammation value.

SUPPLEMENTARY FIGURE 2 Correlation analysis of serum FGF2 and PBIIs in HCs. NLR: neutrophil-to-lymphocyte ratio, dNLR: derived neutrophil-to-lymphocyte ratio, MLR: monocyte-to-lymphocyte ratio, NMLR: neutrophil-to-monocyte-plus-lymphocyte ratio, PLR: platelet-to-lymphocyte ratio, SII: systemic immune inflammatory index, SIRI: systemic inflammatory response index, PIV: pan-immune-inflammation value.

SUPPLEMENTARY FIGURE 3 Comparison of serum FGF2 to PBIIs ratios between ALS patients with bulbar onset and spinal onset. NLR: neutrophil-to-lymphocyte ratio, dNLR: derived neutrophil-to-lymphocyte ratio, MLR: monocyte-to-lymphocyte ratio, NMLR: neutrophil-to-monocyte-plus-lymphocyte ratio, PLR: platelet-to-lymphocyte ratio, SII: systemic immune inflammatory index, SIRI: systemic inflammatory response index, PIV: pan-immune-inflammation value.

SUPPLEMENTARY FIGURE 4 Comparison of serum FGF2 to PBIIs between ALS patients with King stage 1-2 and King stage 3-5. NLR: neutrophil-to-lymphocyte ratio, dNLR: derived neutrophil-to-lymphocyte ratio, MLR: monocyte-to-lymphocyte ratio, NMLR: neutrophil-to-monocyte-plus-lymphocyte ratio, PLR: platelet-to-lymphocyte ratio, SII: systemic immune inflammatory index, SIRI: systemic inflammatory response index, PIV: pan-immune-inflammation value.

SUPPLEMENTARY FIGURE 5 Correlation analysis of FGF2 to PBIIs ratios and age of onset in ALS patients. NLR: neutrophil-to-lymphocyte ratio, dNLR: derived neutrophil-to-lymphocyte ratio, MLR: monocyte-to-lymphocyte ratio, NMLR: neutrophil-to-monocyte-plus-lymphocyte ratio, PLR: platelet-to-lymphocyte ratio, SII: systemic immune inflammatory index, SIRI: systemic inflammatory response index, PIV: pan-immune-inflammation value.

SUPPLEMENTARY FIGURE 6 Correlation analysis of FGF2 to PBIIs ratios and disease duration in ALS patients. NLR: neutrophil-to-lymphocyte ratio, dNLR: derived neutrophil-to-lymphocyte ratio, MLR: monocyte-to-lymphocyte ratio, NMLR: neutrophil-to-monocyte-plus-lymphocyte ratio, PLR: platelet-to-lymphocyte ratio, SII: systemic immune inflammatory index, SIRI: systemic inflammatory response index, PIV: pan-immune-inflammation value.

SUPPLEMENTARY FIGURE 7 Correlation analysis of FGF2 to PBIIs ratios and ALSFRS-R score in ALS patients. NLR: neutrophil-to-lymphocyte ratio, dNLR: derived neutrophil-to-lymphocyte ratio, MLR: monocyte-to-lymphocyte ratio, NMLR: neutrophil-to-monocyte-plus-lymphocyte ratio, PLR: platelet-to-lymphocyte ratio, SII: systemic immune inflammatory index, SIRI: systemic inflammatory response index, PIV: pan-immune-inflammation value.

SUPPLEMENTARY FIGURE 8 Correlation analysis of FGF2 to PBIIs ratios and △ALSFRS-R in ALS patients. NLR: neutrophil-to-lymphocyte ratio, dNLR: derived neutrophil-to-lymphocyte ratio, MLR: monocyte-to-lymphocyte ratio, NMLR: neutrophil-to-monocyte-plus-lymphocyte ratio, PLR: platelet-to-lymphocyte ratio, SII: systemic immune inflammatory index, SIRI: systemic inflammatory response index, PIV: pan-immune-inflammation value.

SUPPLEMENTARY FIGURE 9 ROC curve analysis for the diagnostic performance of FGF2 and FGF2 to PBIIs ratio. ROC: Receiver operating characteristic, FGF2: fibroblast growth factor 2, NLR: neutrophil-to-lymphocyte ratio, dNLR: derived neutrophil-to-lymphocyte ratio, MLR: monocyte-to-lymphocyte ratio, NMLR: neutrophil-to-monocyte-plus-lymphocyte ratio, PLR: platelet-to-lymphocyte ratio, SII: systemic immune inflammatory index, SIRI: systemic inflammatory response index, PIV: pan-immune-inflammation value.

## Glossary

ALS: amyotrophic lateral sclerosis
TDP-43: TAR DNA binding protein 43 (TDP-43)
BDNF: brain-derived neurotrophic factor
FGF2: fibroblast growth factor 2
PBIIs: peripheral blood inflammatory indicators
ELISA: enzyme-linked immunosorbent assay
NLR: neutrophil-to-lymphocyte ratio
PLR: platelet-to-lymphocyte ratio
SII: systemic immune inflammatory index
BMI: body mass index
HCs: health controls
ALSFRS-R: ALS Functional Rating Scale-Revised
dNLR: derived neutrophil-to-lymphocyte ratio
MLR: monocyte-to-lymphocyte ratio
NMLR: neutrophil-to-monocyte-plus-lymphocyte ratio
SIRI: systemic inflammatory response index
PIV: pan-immune-inflammation value
FDR: false discovery rate
ROC: receiver operating characteristic
CI: confidence interval
HRE: hexanucleotide repeat expansion
DPRs: dipeptide repeat proteins
GDNF: glial cell line-derived neurotrophic factor
